# PVDF-Modified Nafion Membrane for Improved Performance of MFC

**DOI:** 10.3390/membranes10080185

**Published:** 2020-08-13

**Authors:** Liping Fan, Junyi Shi, Yaobin Xi

**Affiliations:** 1 College of Information Engineering, Shenyang University of Chemical Technology, Shenyang 110142, China; 2 College of Environment and Safety Engineering, Shenyang University of Chemical Technology, Shenyang 110142, China; sjy1285506862@163.com (J.S.); xiyaobin1@163.com (Y.X.)

**Keywords:** microbial fuel cells, membrane modification, PVDF, acetone

## Abstract

Low power production and unstable power supply are important bottlenecks restricting the application of microbial fuel cells (MFCs). It is necessary to explore effective methods to improve MFC performance. By using molasses wastewater as fuel, carbon felt as an electrode, and the mixture of K_3_[Fe(CN)_6_] and NaCl as a catholyte, an MFC experimental system was set up to study the performance of MFCs with three different proton exchange membranes. A Nafion membrane was used as the basic material, and polyvinylidene fluoride (PVDF) and acetone-modified PVDF were used to modify it, respectively. The experimental results show that a PVDF-modified membrane can improve the water absorption effectively and, thus, make the MFC have greater power generation and better wastewater treatment effect. The acetone-modified PVDF can further improve the stability of output power of the MFC. When the acetone-modified PVDF was used to modify the Nafion membrane, the steady output voltage of the MFC was above 0.21 V, and the Chemical Oxygen Demand (COD) removal rate for molasses wastewater was about 66.7%, which were 96.3% and 75.1% higher than that of the MFC with the ordinary Nafion membrane. Membrane modification with acetone-modified PVDF can not only increase the output voltage of the MFC but also improve the stability of its output electrical energy.

## 1. Introduction

Energy shortage and environmental pollution have become two major problems restricting the sustainable development of human beings. Due to overexploitation, traditional fossil fuels have been nearly exhausted, and the worldwide competition for resources is on the rise. In addition, the exploitation of fossil fuels damages land and villages and pollutes water resources, and the various gases and solid wastes produced in the process of using fossil fuels also pollute the environment. Developing clean energy to replace fossil fuel is an effective way to solve the environmental and energy problems, so it has gradually become the focus of people’s attention and the key development field of various countries [1,2,3].

Microbial fuel cells (MFCs) are a kind of biological reaction device that uses microbial metabolism to convert chemical energy in organic matter into electrical energy directly. An MFC uses organic waste as fuel to generate electric energy in the process of waste treatment, and almost no harmful substances are produced in the process of electricity generation. It can be used not only as a power generation device to solve energy problems but also as a waste disposal device to solve environmental pollution problems. Therefore, it is regarded as an efficient, low-consumption, clean, and environmentally sustainable energy [4,5,6].

Due to the influence of low power generation, high cost, and unstable work, the popularization and application of MFCs is still restricted [7]. The main factors affecting the performance of MFCs include microbial population, exchange membrane, electrode, internal and external resistance, substrate concentration, electrode spacing, etc. [8,9,10,11]. Researchers have tried various methods to improve the performance of MFCs. The common methods include the fabrication of new electrode materials, the development of a more efficient exchange membrane, the modification of carbon-based electrode materials and Nafion membrane, the cultivation of more efficient electrogenic bacteria, and so on. When a potassium dichromate (K_2_Cr_2_O_7_)-modified carbon cloth anode was used in an MFC, the power generation performance and wastewater treatment effects were significantly improved [12]. Au-g-C_3_N_4_ nanostructures have high electron transfer efficiency and can be used as MFC electrodes to improve electrochemical performance [13]. Ag@g-C_3_N_4_ NSs, which was fabricated by an innovative and simple biogenic approach, can produce highly reactive oxygen species (ROS) [14], if it is used as the cathode or catalyst of MFCs, the reaction rate of the cathode chamber can be accelerated. The MFCs using a Mo_2_C/CCT (molybdenum carbide nanoparticles-modified carbonized cotton textile) anode can produce higher power density [15]. Some research found that the use of mutualistic interaction of yeast and bacteria could increase the performance of the MFCs [16].

Among all these factors that affect MFC performance, the proton exchange membrane is considered to be the most important factor affecting the operation effect of MFC. A proton exchange membrane is an important part to separate the anode chamber and cathode chamber and transfer protons from anode to cathode; moreover, the cost of the membrane accounts for more than 38% of the total cost of MFCs [17,18], so it is also one of the important factor affecting the promotion and application of MFCs.

Proton exchange membranes have been widely used in MFCs because of their advantages of high conductivity and low internal resistance [19,20,21]. The most commonly used proton exchange membrane in MFCs is perfluorosulfonic acid membrane, such as a Nafion membrane produced by the DuPont Company. The main advantages of Nafion membranes are their high proton conductivity, good chemical stability, and mechanical properties, but it still has some drawbacks such as high oxygen permeability, poor thermal stability, biological deposition, and serious water loss at high temperature, which limits the performance of MFCs [22]. In order to solve these problems, it is an effective and recognized way to improve the performance of MFCs by membrane modification.

Polyvinylidene fluoride (PVDF) is a pure thermoplastic fluoropolymer. PVDF not only has the advantages of good toughness, low friction coefficient, corrosion-resistant, and anti-aging but also has the characteristics of low density, low impedance, and good chemical stability [23,24,25,26,27]. On the other hand, compared with a Nafion membrane itself, PVDF is a relatively cheap material. Modifying a Nafion membrane by PVDF can improve the water retention and its adaptability to temperature and humidity at a very low cost [28] and then increase the electricity-generating capacity and the wastewater treatment efficiency of MFCs, so the overall cost performance of an MFC system is improved.

In this paper, PVDF particles and a kind of modified PVDF solution were used to modify the proton exchange membrane, and the effects of PVDF and modified PVDF on the power generation performance and water purification effect of MFCs were studied. In addition, a group of experiments was added to study the effect of carbon cloth and carbon felt electrodes on the operation of MFCs under the same other conditions.

## 2. Materials and Methods

### 2.1. Composition of the Experimental System

**Figure 1:**
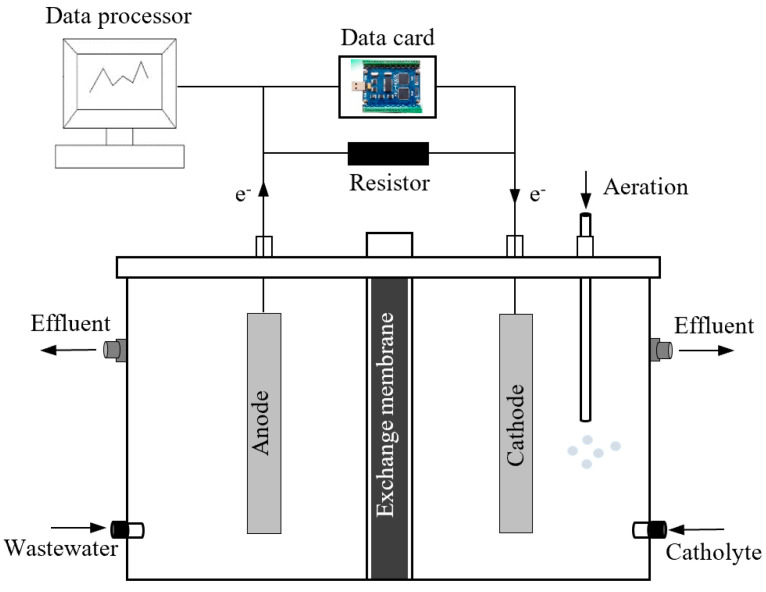
Schematic diagram of the MFC experiment system.

The experimental system consists of three parts, namely, a dual chamber MFC, an external load and a data acquisition system (Figure 1). A dual chamber MFC is mainly composed of a cathode chamber, anode chamber, proton exchange membrane, cathode, and anode. The reaction chambers were made of plexiglass. The cathode chamber and anode chamber are separated by a proton exchange membrane (Nafion 117; DuPont Co., Wilmington, DE, USA). The volume of both the cathode and anode reaction chambers was 500 mL. Both the anode and cathode materials were carbon felt with surface areas of 4 cm × 4.5 cm. The anode and the cathode were connected with external load through wires to form a complete closed circuit. The voltage generated by the MFC was collected through the data acquisition card (MPS-010602; Morpheus Electronics Technology Co., Beijing, China), recorded every 60 s, and transmitted to a computer through USB interface for storage, processing, and display.

### 2.2. Experimental Materials and Pretreatment

The anolyte of the MFC for experiment was molasses wastewater, which was prepared by mixing the following chemicals: 3.13 g/L NaHCO_3_, 0.31 g/L NH_4_Cl, 6.338 g/L NaH_2_PO_4_·H_2_O, 0.13 g/L KCl, 0.2 g/L MgSO_4_·7H_2_O, 0.015 g/L CaCl_2_, 3 g/L brown sugar, 0.01 g/L MnSO_4_·H_2_O, and 6.8556 g/L Na_2_HPO_4_·12H_2_O.

The catholyte used in the experiment was a mixture of potassium ferricyanide (K_3_[Fe(CN)_6_]) and sodium chloride (NaCl). 0.2 mol/L K_3_[Fe(CN)_6_] (32.9 g) was dissolved in a phosphoric acid buffer solution (PBS) in a 500 mL volumetric flask; we put 0.4 mol/L NaCl (11.7 g) into another 500 mL volumetric flask; then the K_3_[Fe(CN)_6_] solution described above were mixed with NaCl solution in a ratio of 1:1 to obtain the catholyte of the MFC. All the reagents used in the experiment were purchased from Damao Co, Tianjin, China.

Many research results show that soil is rich in microorganisms [29,30]. So the electrogenic bacteria used in the anode chamber were obtained by culturing some soil. The soil was collected around some trees in the campus. Under anaerobic conditions, the prepared simulated molasses wastewater and the collected soil were put into the culture flask together, then some trace elements (such as carbon, nitrogen, phosphorus, etc.) needed for microbial growth were added, and then the culture flask was put into the biochemical incubator and domesticated at 30 °C for about 3 days. When the sludge in the culture flask was suspended as flocculate, the culture of microorganisms was considered to be successful.

### 2.3. Preparation of Membranes

We boiled the proton exchange membrane (Nafion 117) in 5% H_2_O_2_ solution for 1 h, then took it out and washed it repeatedly with deionized water to remove organic impurities; then boiled it in 1 mol/L H_2_SO_4_ for 1 h to remove metal ion impurities; we then took it out and washed it repeatedly with deionized water again and boiled it for 1 h with deionized water to remove the residual H_2_SO_4_. The pretreated proton exchange membrane was put into deionized water for standby.

(1) Preparation of PVDF and modified PVDF solution

DMF (*N*,*N*-dimethylformamide) is a widely used excellent organic solvent with good solubility and chemical stability for a variety of organic and inorganic compounds [31]. Therefore, we used DMF as solvent to make the PVDF solution.

12.47 g PVDF was dissolved in 250 mL DMF solution and stirred for 2 h at room temperature to obtain white PVDF solution.

11.21 g PVDF was dissolved in 250 mL mixture of DMF and acetone with a volume ratio of 4:6 and stirred continuously for 2 h at room temperature, and then the modified PVDF solution with milky white color was obtained.

(2) Preparation of composite membrane

The Nafion membrane with a size of 10 cm × 10 cm was immersed in a methanol solution with a volume ratio of 3:1 for 1 h to make the membrane surface swell, and thus the PVDF particles can better adhere to it; then the Nafion membrane was placed in the PVDF solution and soaked at a constant temperature of 60 °C for 5 min; then it was taken out and dried and placed in a vacuum drying oven to dry at a constant temperature of 60 °C for 15 min. Repeating the above operations for four or five times, PVDF–Nafion composite membrane was obtained.

We immersed another Nafion membrane in the modified PVDF solution, treated the Nafion membrane in the same way as above, and finally, the modified PVDF–Nafion composite membrane was prepared [32].

### 2.4. Analysis Method

The performance analysis of an MFC mainly includes power generation performance and wastewater treatment effect.

The current density value is generally used to evaluate the power generation performance. The output current *I* of the MFC can be calculated by Ohm’s law according to the measured voltage value *U*, that is:

(1) I=U/R

in which *U* is the output voltage of the MFC measured by data acquisition card and *R* is the external load resistance. In the experiment, *R* is always set at 500 Ω.

Then the generating current density *I_A_* of the MFC can be obtained by calculating the following formula:

(2) $$I_{A}=\frac{I}{A}=\frac{U}{AR}$$

where *A* is the anode surface area of the fuel cell.

Wastewater treatment effect is also an important index of MFC performance. In MFCs, microorganisms degrade organics to generate electricity; meanwhile, the wastewater that is used as an anolyte (or fuel) of the MFC can also be purified well. In order to analyze the wastewater treatment effect of the MFC, the influent COD and effluent COD of the anode chamber were measured by a LH-NP2 type COD rapid detector (Lohand biological company, Dalian, China), and then the COD removal rate of the MFC was calculated, according to which the wastewater treatment effect of the MFC was analyzed subsequently.

Through the physical structure and morphology of the material, the effect of the membrane modification can be analyzed more deeply in essence. Scanning electron microscope (SEM) (JSM-6360LV; JEOL Co., Tokyo, Japan) analysis of the exchange membrane before and after modification was carried out and electrochemical impedance spectroscopy (EIS) (CHI660E, CH Instrument Co., Shanghai, China) analysis of the MFC was also provided in this experiment.

## 3. Results and Discussion

### 3.1. Comparison of Power Generation Performance

Four MFC experimental systems with the same basic configuration were run simultaneously to guarantee that the other experimental conditions are the same except for the comparison items, so as to obtain a good comparison effect.

**Figure 2:**
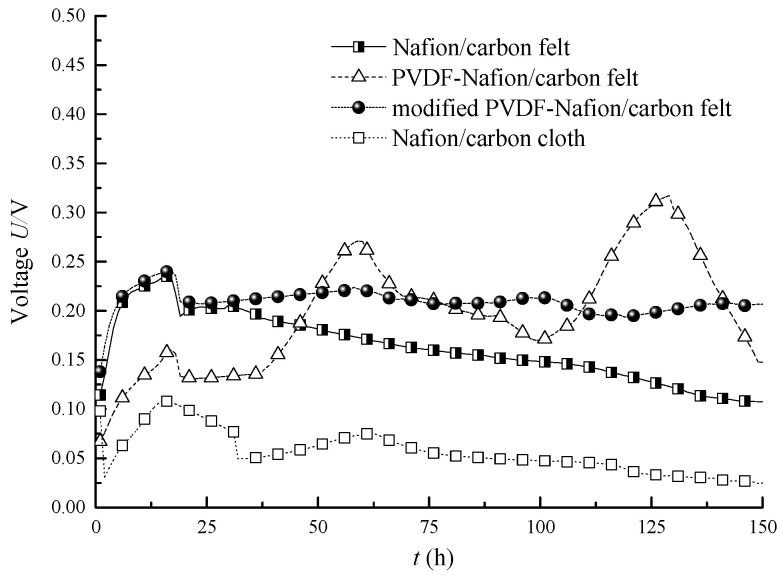
Output voltage curves of MFCs under different conditions.

Firstly, the influence of different electrodes on the power generation performance of the MFC with the same Nafion membrane was analyzed. Figure 2 shows the output voltage curves of MFCs with different electrodes and different exchange membranes. It can be seen from the figure that the power generation capacity of MFC with carbon cloth electrode was obviously different from that of the MFC with a carbon felt electrode. When both the exchange membranes of the MFCs are common Nafion membranes, the steady-state output voltage of the MFC with a carbon cloth electrode was about 0.025 V, while the steady-state output voltage of the MFC with a carbon felt electrode was about 0.107 V. That is to say, the output voltage of the MFC with a carbon felt electrode is about 328% higher than that of MFC with a carbon cloth electrode. This is mainly due to the fact that the carbon felt has a larger specific surface area and richer and finer pores than the carbon cloth. Therefore, it is more beneficial for microorganisms to be adhered to its surface and, thus, increases the growth space of the anode biofilm and improves the electron transfer of the whole system. In view of the above experimental results, carbon felt was used as the electrode of the MFC in the later experiments.

It can also be seen from Figure 2 that the output voltage of the MFC with a PVDF-modified Nafion membrane was significantly higher than that of the MFC with a common Nafion membrane. This is mainly because the exchange membrane modified by PVDF improves the water retention performance, so the water absorption capacity and proton conductivity are improved. The introduction of organic materials is also effective to improve the conductivity of the exchange membrane. However, when the exchange membrane was modified by general PVDF, although the output voltage was significantly higher than that of the MFC with a normal Nafion membrane, the output voltage fluctuated so much that it was difficult to stabilize. Although the maximum voltage can reach 0.3 V, the output voltage cannot be kept at a high value stably and drops sharply after reaching the peak value; in the early stage of its operation, the start-up voltage of the MFC is relatively low. Low starting voltage and fluctuating output are major problems affecting practical application. Therefore, the improved PVDF was used to modify the membrane to further improve the power generation stability of the MFC. The stability of the output voltage of the MFC with a modified PVDF–Nafion composite membrane was improved obviously; in this case, the steady state output voltage was about 0.21 V, which was 96.3% higher than that of the MFC with a common Nafion membrane.

The stability of the output voltage of the MFC with a modified PVDF–Nafion membrane has been improved significantly, mainly because the acetone solvent accelerated the rate of acceptance of PVDF by the Nafion membrane; in addition, the porous and disordered structure on the surface of the Nafion membrane makes PVDF easier to adhere to, making the proton transmission performance of the modified Nafion membrane more stable, thus improving the stability of the output electric energy of the MFC.

In order to compare the power generation capacity of MFCs in the several different conditions more effectively, the output electrical energy values of the MFC with four different conditions were calculated and compared. The formula that links energy *W* and power *P* is:

(3) W=Pt

The power can be calculated by using the Joule’s law equation, so the energy can be described by:

(4) $$W=UIt=\frac{U^{2}}{R}t$$

Since the output voltage of the MFC is time-varying, the output electrical energy within 0 h to 150 h can be expressed as:

(5) $$W=\int_{0}^{150}\frac{u^{2}}{R}dt$$

**Figure 3:**
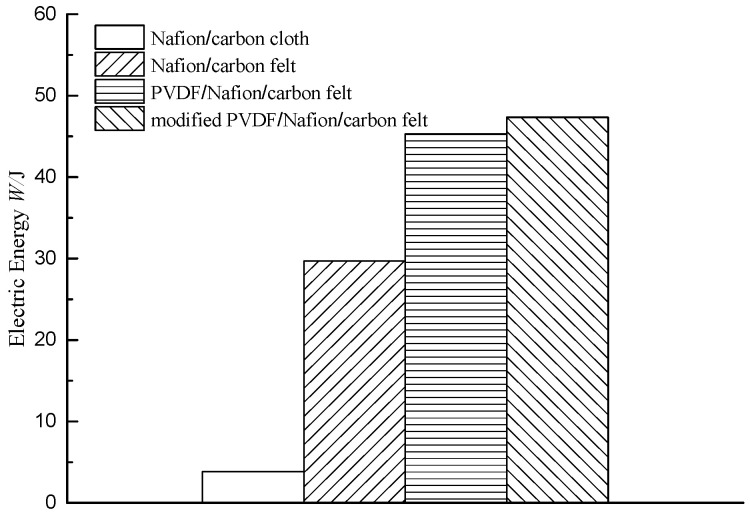
Electrical energy of MFCs with different conditions.

Figure 3 shows the electrical energy values generated by the four MFCs with different conditions. It can be seen that the MFC with a carbon cloth electrode produced much less electrical energy than the MFC with a carbon felt electrode. The output electrical energy of the MFC with the common Nafion membrane and carbon cloth electrode was 3.83 J. When a carbon felt electrode was used, the electrical energy generated by the MFCs using the common Nafion membrane, the PVDF–Nafion composite membrane, and the modified PVDF–Nafion membrane were 29.7 J, 45.3 J, and 47.4 J, respectively. The electrical energy produced by the MFC with the common Nafion membrane was significantly lower than that of the MFC with the two composite membranes. This shows that the PVDF-modified proton exchange membrane significantly enhances the power generation capacity of MFCs. The electrical energy of the MFC with modified PVDF membrane was slightly higher than that of the MFC with a general PVDF-modified membrane. This shows that the use of the modified PVDF membrane can not only significantly enhance the power generation stability of the MFC but also improves the power generation performance of MFC. Therefore, modification of the exchange membrane by modified PVDF has a good effect on improving the overall performance of the MFC.

In order to further analyze the effect of the modified PVDF on the stability of the MFC, the standard deviation of the output current density of the MFC was calculated.

The steady-state value of the current density that characterizes the power supply capacity of the MFC can be obtained according to the calculation formula of the average value:

(6) $$\mu =\frac{X_{1}+X_{2}+\ldots +X_{n}}{n}$$

where *μ* is the average value of current density (A/m^2^); *X_i_* is the value of current density of the *i*th sample (A/m^2^); *n* is the number of samples.

The power supply stability of the MFC can be characterized by standard deviation, i.e.,

(7) $$\sigma =\sqrt{\frac{1}{n}\sum_{i=1}^{n}(X_{i}-\mu {)}^{2}}$$

where *σ* is the standard deviation of the output current density (A/m^2^).

Standard deviation is a measure of the degree of dispersion of the average of a set of data. A larger standard deviation means that there is a larger difference between the sample data and its average value, while a smaller standard deviation means that the sample data is closer to the average value. The smaller the standard deviation, the smaller the volatility and the better the stability [33,34].

**Figure 4:**
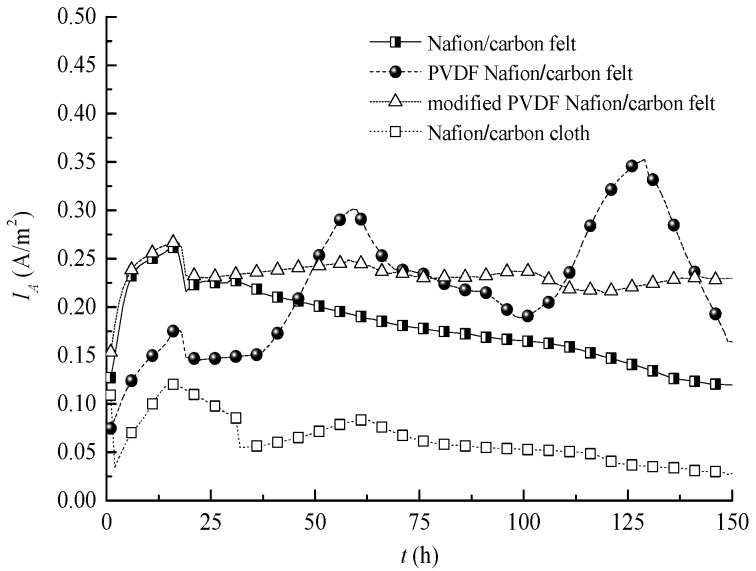
Current density curves of MFCs in different conditions.

Figure 4 shows the current density curves of the MFC in different conditions. It can be seen that the steady-state values of the current density of the MFC with the PVDF–Nafion composite membrane and the modified PVDF–Nafion membrane were 0.219 A/m^2^ and 0.233 A/m^2^, respectively. Although there was only little difference between the two, it can be seen from Table 1 that the standard deviation values of the current density of the MFC with the PVDF composite membrane and the MFC with the modified PVDF composite membrane were 0.062 A/m^2^ and 0.013 A/m^2^, respectively, and the standard deviation of the current density of the MFC with the modified PVDF composite membrane was significantly lower than that of the MFC with the common PVDF composite membrane. This means that the modified PVDF membrane significantly improves the stability of the output power of the MFC. The modified PVDF membrane can effectively overcome the serious fluctuation of the output electrical energy of the MFC caused by the conventional PVDF-modified membrane, which not only increases the output voltage of the MFC, but also improves the stability of its output electrical energy. This is of great significance for the practical application of MFCs.

### 3.2. SEM Analysis

**Figure 5:**
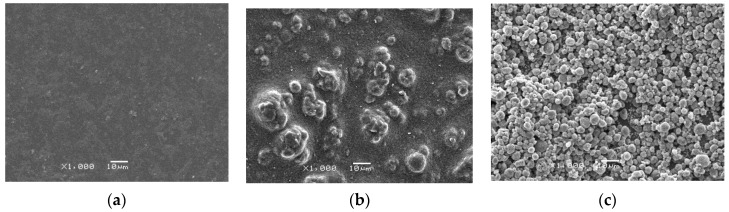
SEM images. (**a**) general Nafion membrane. (**b**) PVDF–Nafion composite membrane. (**c**) modified PVDF–Nafion composite membrane.

The SEM images of the three different proton exchange membranes are shown in Figure 5. It can be seen that the surface of the conventional Nafion membrane had no obvious morphological characteristics except for some sporadic impurities, and it seems to be a compact plane shape as a whole. The surface morphology of the PVDF-modified proton exchange membrane was rough and mossy, and there were many granular and agglomerated substances protruding from the surface, which should be due to the uneven distribution of the modifier. Moreover, the white PVDF particles on the surface of the membrane were sparse and uneven, which may be the main reason for the instability of the power generation performance. However, the surface of the acetone-modified PVDF–Nafion composite membrane was covered by many regular granular polymers, and more pores were formed, which is more conducive to proton conduction. From the overall point of view of the composite membrane, the distribution of PVDF white particles was dense and uniform, indicating that PVDF particles were well attached to Nafion membrane, thus improving the power production and output stability of MFCs. This indicates that the modified PVDF can bring a better modification effect on the proton exchange membrane than ordinary PVDF.

### 3.3. Hydroscopicity Analysis of Membrane

The water absorption performance of the proton exchange membrane is directly related to the power generation performance of MFC. When the water content of the membrane is high, the migration coefficient of the proton is higher than that of water, and the conductivity is also high. Therefore, the higher the water absorption of the membrane, the better its conductivity. To compare the hydroscopicity of the three different membranes used in the experiment, the water absorption of the three membranes was calculated, respectively, by measuring the weight of the membrane before and after hydration [35,36].

Firstly, the membrane was immersed in deionized water at room temperature for 24 h. Then the membrane surface was dried with the filter paper immediately after taking it out, and then was weighed and marked as wet weight of the membrane *W*_1_. The membrane was then placed in an oven at 80 °C and baked for 4 h and, then, was taken out and cooled to room temperature, weighed, and recorded as dry weight *W*_0_. Then the water absorption of the membrane can be calculated as:

(8) $$\Delta W(\%)=\frac{W_{1}-W_{0}}{W_{0}}\times 100\%$$

The water absorption rates of the three different membranes are shown in Table 2.

It can be seen from Table 2 that the water absorption rate of the PVDF membrane was 12%, while that of the modified PVDF membrane was 11.8%, which was 87.2% and 84.1% higher than that of the conventional Nafion membrane, respectively. The water absorption rate of the modified PVDF composite membrane was basically the same as that of the PVDF membrane. This shows that the conductivity of the two kinds of PVDF-modified membranes was approximately the same, which further proves the above experimental results.

### 3.4. Analysis of EIS

**Figure 6:**
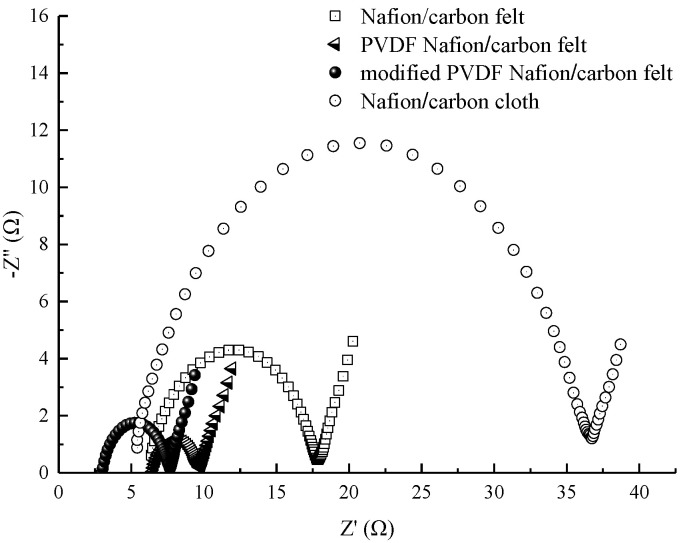
Electrochemical impedance spectra of MFCs under different conditions.

EIS can reflect the dynamic process, mechanism, and impedance information of the whole MFC system. The internal resistance and its own reaction kinetics process are also important factors affecting the operation effect of the MFC. The Nyquist plots of the four MFCs under different operating conditions are shown in Figure 6. Each curve is composed of two parts, namely, the semicircle in the high frequency region and the straight line in the low frequency region. The value of the left intersection of the semicircle and the horizontal axis is the solution internal resistance *R*_s_; the diameter of the semicircle is the charge transfer impedance *R*_ct_ caused by redox reaction on the electrode surface. The straight line in the low frequency region represents the Warburg impedance, which reflects the diffusion degree of ions in the electrode [37,38,39].

It can be seen from Figure 6 that the charge transfer impedance *R*_ct_ of the MFC with conventional Nafion membrane and carbon felt anode is about 10 Ω, which is significantly greater than that of PVDF–Nafion membrane and modified PVDF–Nafion membrane. The test results show that the MFC with the conventional Nafion membrane has a large polarization internal resistance, while the MFC constructed by the PVDF-modified proton exchange membrane reduces the polarization internal resistance. This may be due to the improvement of proton transfer efficiency and electrochemical reaction efficiency of the whole system by PVDF-modified proton exchange membrane, thus reducing the polarization internal resistance. At the same time, it also can be seen that the solution internal resistance *R*_s_ of MFC with the ordinary PVDF–Nafion membrane and the MFC with the modified PVDF–Nafion membrane were about 6.5 Ω and 2.6 Ω, respectively. Compared with the general PVDF-modified membrane, the modified PVDF membrane can make the MFCs have a lower solution resistance. Therefore, the modified PVDF is a more suitable modifier for improving the performance of proton exchange membranes.

What is more, it can also be seen from the comparative experimental results that the solution internal impedance of the MFC with the carbon cloth electrode was almost the same as that of the MFC with the carbon felt electrode, but the charge transfer impedance was quite different. The charge transfer impedance of the MFC with the carbon cloth electrode was about 30 Ω, which was about three times that of the MFC with the carbon felt electrode. This further indicates that carbon felt is more suitable for use as an electrode of MFCs than carbon cloth.

**Figure 7:**
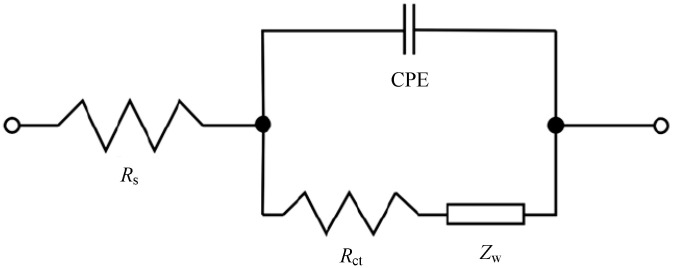
The equivalent circuit of MFCs under different conditions.

To obtain more detailed impedance information, the impedance data were fitted in Zview (Scribner Associates Inc., Southern Pines, NC, USA). The equivalent circuit is shown in Figure 7, where *Z*_w_ is the Warburg impedance reflecting the resistance encountered by the reactants in the process of diffusion in solution; CPE is a constant phase element in which the double layer impedance is represented.

The impedance data obtained by equivalent circuit fitting are listed in Table 3. It can be seen that the Warburg impedance changed little in several different cases, but the solution impedance of acetone-modified PVDF composite membrane was the smallest.

### 3.5. Water Quality Analysis

When molasses wastewater was used as an anolyte of the MFC, the COD values of influent and effluent of MFCs with different proton exchange membranes are shown in Table 4. It can be seen that the COD removal rate of the MFC with conventional Nafion membrane was 38.1%, while the COD removal rates of the MFC with the PVDF–Nafion composite membrane and the MFC with the modified PVDF–Nafion composite membrane were 63% and 66.7%, respectively, which were 65.4% and 75.1% higher than that of MFC with ordinary Nafion membrane. Therefore, when the molasses wastewater was used as the fuel of MFC, the molasses wastewater had also been well purified and treated while the organic matter was degraded by the microorganisms, and the use of the modified PVDF membrane further improved the water purification effect of the MFC. This indicates that MFCs can not only degrade organic matter to produce electric energy but also purify the wastewater as feed. In particular, when the proton exchange membrane was loaded by the modified PVDF, the purification effect of the MFC was significantly improved. 

Molasses wastewater is a kind of wastewater with high pollutant concentration, which is rich in sugars, proteins, amino acids, vitamins, and other organic matter. Molasses wastewater, which has high concentrations of COD and complex components, could cause serious environmental pollution in the ecosystem [40]. At present, the COD removal rate of the existing molasses wastewater treatment methods is generally not very high. For example, the COD removal rate of molasses wastewater by UV photolysis method was only 2%; the COD removal rate of a complex hybrid system was only about 15% [41,42], and the COD removal rate of molasses wastewater by anaerobic tank with better treatment effect was about 46.0% [43]. Therefore, it is an alternative method to use MFCs to treat molasses wastewater. Most importantly, MFCs can generate electric energy while treating wastewater, which provides an effective way to solve the dual pressure of environmental pollution and energy crisis at the same time. So, MFCs are a promising technology for power generation and wastewater treatment.

## 4. Conclusions

Using PVDF particles to modify the Nafion membrane can improve the power generation performance and water purification effect of the MFC effectively. The membrane modified by the modified PVDF solution can further improve the adhesion of PVDF on the membrane and, thus, improve the power generation stability of the MFC. When the modified PVDF–Nafion composite membrane was used, the performance of MFC in power generation capacity, power supply stability, sewage treatment effect, and other aspects were significantly improved. In this case, the output voltage of MFC can be basically stable at 0.21 V in most of the running time, which was 96.3% higher than that of the MFC with the conventional Nafion membrane; the COD removal rate of molasses wastewater can reach 66.7%, which is 75.1% higher than that of the MFC with the ordinary Nafion membrane. Therefore, PVDF is a valuable membrane modification material, which can improve the performance of MFCs.

In addition, although the water purification effect of the MFC with the carbon felt electrode is almost the same as the MFC with the carbon cloth electrode, the electricity production capacity of the MFC with the carbon felt electrode is much higher than that of the MFC with the carbon cloth electrode. Therefore, carbon felt is a better electrode for the MFC than the carbon cloth.

## Figures and Tables

**Table 1:** Steady-state values and their standard deviations of current density.

	Nafion/Carbon Cloth	Nafion/Carbon Felt	PVDF–Nafion/Carbon Felt	Modified PVDF–Nafion/Carbon Felt
Steady-state value (A/m^2^)	0.062	0.181	0.219	0.233
Standard deviation (A/m^2^)	0.023	0.037	0.062	0.013

**Table 2:** Water absorption rate of different membranes.

	Nafion/Carbon Felt	PVDF–Nafion/Carbon Felt	Modified PVDF–Nafion/Carbon Felt
Wet weight *W*_1_ (g)	3.65	3.92	3.87
Dry weight *W*_0_ (g)	3.43	3.50	3.46
Water absorption rate Δ*W* (%)	6.41	12.00	11.80

**Table 3:** Impedance data obtained by equivalent circuit fitting.

	Nafion/Carbon Felt	PVDF–Nafion/Carbon Felt	Modified PVDF–Nafion/Carbon Felt	Nafion/Carbon Cloth
*R*_s_/Ω	6.191	6.518	3.162	4.977
*R*_ct_/Ω	11.36	2.897	4.138	31.38
*Z*_w_/(Ω)	0.7962	0.6684	0.8775	0.7758

**Table 4:** Influent and effluent COD of MFCs with different membranes (with carbon felt electrode).

	Nafion	PVDF–Nafion	Modified PVDF–Nafion
Influent (mg/L)	3663	3663	3663
Effluent (mg/L)	2266	1354	1220
COD removal rate (%)	38.1	63.0	66.7
